# Feasibility and Safety of Operating Room Extubation After Minimally Invasive Cardiac Valve Surgery: A Systematic Review and Meta-Analysis

**DOI:** 10.3390/jcdd13080368

**Published:** 2026-08-04

**Authors:** Dimitrios E. Magouliotis, Serge Sicouri, Vasiliki Androutsopoulou, Massimo Baudo, Vanesa Brecher, Dimitrios V. Avgerinos, Thanos Athanasiou, Basel Ramlawi

**Affiliations:** 1 Department of Cardiac Surgery Research, Lankenau Institute for Medical Research, Wynnewood, PA 19096, USA; sicouris@mlhs.org (S.S.); massimo.baudo@icloud.com (M.B.); vb4075@pcom.edu (V.B.); ramlawib@mlhs.org (B.R.); 2 Department of Cardiothoracic Surgery, Faculty of Medicine, University of Thessaly, 41110 Larissa, Greece; androutsopoulouvasiliki@uth.gr; 3 Department of Cardiac Surgery, Onassis Cardiac Surgery Center, 17674 Athens, Greece; davgerinos@gmail.com; 4 Department of Surgery and Cancer, Imperial College London, London SW7 2AZ, UK; t.athanasiou@imperial.ac.uk; 5 Department of Cardiac Surgery, Lankenau Heart Institute, Main Line Health, Wynnewood, PA 19096, USA

**Keywords:** operating room extubation, OR extubation, on-table extubation, fast-track cardiac anesthesia, minimally invasive valve surgery

## Abstract

Background: Minimally invasive cardiac valve surgery has emerged as a preferred approach in selected patients, yet optimal postoperative extubation timing remains debated. This systematic review and meta-analysis examined clinical outcomes associated with extubation in the operating room (OR) versus the intensive care unit (ICU) among adult patients undergoing minimally invasive cardiac valve surgery. Methods: The study was conducted according to PRISMA guidelines. A single unit of analysis was applied throughout. Pooled odds ratios were computed with the Mantel–Haenszel random-effects method; where a study reported only a matched or covariate-adjusted estimate, that estimate was reserved for a prespecified sensitivity analysis using the generic inverse-variance method. Results: Five observational studies (2023–2025) including 1101 OR-extubated and 899 ICU-extubated patients from high-volume centers with fast-track or enhanced recovery pathways were included. OR extubation was associated with lower odds of reintubation (OR 0.40; 95% CI 0.24–0.69; *I*^2^ = 0%), postoperative delirium (OR 0.47; 95% CI 0.31–0.72; *I*^2^ = 0%), and pneumonia (OR 0.30; 95% CI 0.16–0.53; *I*^2^ = 0%). No significant differences were observed for new-onset atrial fibrillation, stroke, or reoperation for bleeding. Thirty-day mortality was reported by four of the five studies and comprised few events (5 of 1043 ORE versus 17 of 645 ICE across the four studies reporting this outcome); given the small number of events, the concentration of deaths in the higher-risk ICU-extubated patients, and the reliance of the pooled estimate on two confounded cohorts, this difference is not interpretable as a treatment effect, and no pooled odds ratio is reported here. Length of stay was consistently shorter after OR extubation but was not pooled because of extreme heterogeneity (*I*^2^ = 96–100%). Sensitivity analyses using adjusted estimates attenuated the associations for reintubation and pneumonia, consistent with substantial confounding by indication. Conclusions: In appropriately selected patients undergoing minimally invasive valve surgery, OR extubation is feasible and is associated with a recovery profile at least comparable to that of ICU extubation. Because extubation location was determined largely by intraoperative and early postoperative stability, these associations should be read as reflecting patient selection rather than a causal benefit of the strategy. The findings support the feasibility of OR extubation in appropriately selected patients at experienced centers and motivate prospective, ideally randomized, evaluation.

## 1. Introduction

Minimally invasive cardiac valve surgery has increasingly replaced conventional sternotomy in specialized centers, driven by its association with reduced surgical trauma, shorter hospitalization, lower transfusion requirements, and faster functional recovery compared with full sternotomy approaches [1,2,3,4]. As surgical access has evolved, perioperative management has similarly undergone substantial refinement, particularly in ventilation strategies and extubation timing. Early extubation after cardiac surgery emerged in the 1990s as a cornerstone of “fast-track cardiac anesthesia” [5,6,7], and subsequent work has demonstrated its safety and potential to reduce intensive care unit (ICU) stay, resource utilization, and postoperative complications.

Enhanced Recovery After Surgery (ERAS) and Enhanced Recovery After Cardiac Surgery (ERACS) protocols have further formalized this evolution by integrating multimodal analgesia, opioid-sparing anesthetic techniques, early mobilization, and structured pathways for extubation [8,9,10,11]. Contemporary fast-track cardiac anesthesia guidance endorses extubation within six hours following adult cardiac surgery when feasible [12]. Nonetheless, whether extubation performed directly in the operating room (OR) yields additional advantages remains a subject of ongoing debate.

Several centers have adopted OR extubation protocols for select cardiac procedures, particularly minimally invasive valve surgery, based on the rationale that reduced surgical trauma, shorter cardiopulmonary bypass times, and optimized anesthetic strategies enhance physiologic readiness for immediate liberation from mechanical ventilation [10,11]. Proposed benefits include decreased intubation duration, reduced incidence of pulmonary complications, avoidance of sedative accumulation, and improved ICU throughput. Despite this, concerns persist. Some clinicians caution that OR extubation may expose patients to higher risks of hemodynamic instability, bleeding, respiratory failure, or the need for reintubation in the early postoperative period [12]. Moreover, evidence across the literature remains heterogeneous, and high-quality comparative data are limited.

As minimally invasive mitral, tricuspid, and aortic valve surgery continues to grow, understanding the impact of extubation strategy on patient outcomes has direct implications for clinical pathways, ICU resource allocation, patient-centered recovery, and hospital costs. While individual observational cohorts suggest potential advantages of OR extubation, including reductions in ICU and hospital length of stay, no comprehensive synthesis has examined its safety and effectiveness across minimally invasive valve procedures.

Therefore, the objective of this systematic review and meta-analysis is to compare the safety and clinical outcomes of OR extubation (ORE) versus ICU extubation (ICE) following minimally invasive cardiac valve surgery. By integrating all available evidence at a single, consistent unit of analysis, we aim to characterize the associations between extubation strategy and postoperative recovery and to clarify the extent to which these associations reflect patient selection rather than a causal treatment effect.

## 2. Materials and Methods

### 2.1. Study Design and Ethical Considerations

This systematic review and meta-analysis was performed in accordance with the PRISMA guidelines [13]. The protocol of the current study was registered in the Open Science Framework (OSF) Registries (registration doi: 10.17605/OSF.IO/HQSD5). Because all data were derived from previously published studies and contained no individual patient identifiers, institutional review board approval was not required.

### 2.2. Literature Search Strategy

A comprehensive search of PubMed, EMBASE, Cochrane CENTRAL, Web of Science, and Scopus was conducted, with the final search being completed on 20 November 2025. Search terms included combinations of “minimally invasive cardiac surgery”, “minimally invasive valve surgery”, “right thoracotomy”, “video-assisted”, “fast-track anesthesia”, “operating room extubation”, “on-table extubation”, “OR extubation”, “immediate extubation”, “same-day extubation”, “ultra-fast-track”, “traditional extubation”, and “ICU extubation”. These synonyms were included specifically to capture earlier reports that might have used pre-2020 terminology for the same technique. The reference lists of all retrieved studies were manually reviewed to identify additional eligible publications.

The inclusion criteria for this meta-analysis were as follows: (1) original clinical studies enrolling at least 10 adult patients, (2) published between 2000 and 2025, (3) written in English, (4) conducted on human subjects undergoing minimally invasive cardiac valve surgery, and (5) reporting comparative outcomes between extubation performed in the operating room and extubation performed in the intensive care unit. No study published before 2023 met these criteria. This is not an artifact of the search terminology: the search deliberately included older synonyms for the technique (for example, immediate, same-day, on-table, and ultra-fast-track extubation), and earlier reports of fast-track or immediate extubation after cardiac surgery either did not perform minimally invasive valve surgery specifically or did not report a direct comparison between operating room and intensive care unit extubation, which are the eligibility requirements here. The concentration of eligible studies in 2023 to 2025 therefore reflects the recent emergence of this specific comparison in the literature rather than an inconsistency in the search period. Duplicate publications were excluded. The kappa coefficient was calculated to assess the level of agreement between the two reviewers. Two independent reviewers (DEM and SS) extracted data from the included studies, and any disagreements were resolved through discussion with the senior author (BR) until consensus was achieved.

### 2.3. Quality and Publication Bias Assessment

To evaluate the methodological rigor of the non-randomized studies included in this review, we used the Newcastle–Ottawa Scale (NOS) [14], which assesses observational studies based on selection of cohorts, comparability of study groups, and adequacy of outcome measurement. Scores range from zero to nine, and studies receiving five or more points were considered to have acceptable quality. We also applied the Risk of Bias in Non-Randomized Studies of Interventions (ROBINS-I) tool [15] to further examine potential sources of bias across included cohorts. As no randomized controlled trials were identified, all quality assessments were performed independently by two reviewers (DEM and SS), with any discrepancies resolved through discussion until agreement was reached.

With fewer than ten studies contributing to any single endpoint, funnel-plot methods and formal statistical tests for small-study effects, including Egger’s test [16], are considered uninformative and were not used to draw conclusions about publication bias. We therefore did not assess publication or small-study bias for any endpoint, and note this as a limitation of the available evidence base. A leave-one-out sensitivity analysis was conducted for all pooled endpoints to assess the robustness of the primary estimates.

### 2.4. Statistical Analysis

All statistical analyses were performed using Review Manager (RevMan), version 5.4.1, developed by the Cochrane Collaboration, London, UK [17]. Dichotomous outcomes, such as reintubation, postoperative complications, and mortality, were pooled using the Mantel–Haenszel method. A random-effects model was applied to accommodate the anticipated clinical and methodological variability among studies. Effect estimates were reported as odds ratios (ORs) with corresponding 95% confidence intervals (CIs). Statistical heterogeneity was quantified using the *I*^2^ statistic, with values of 25%, 50%, and 75% interpreted as low, moderate, and high heterogeneity, respectively [17]. A *p*-value < 0.05 was considered statistically significant.

A single, explicit unit of analysis was applied across all pooled endpoints. For the primary analysis, each study contributed its full reported cohort of ORE and ICE patients [18,19,20,21]. Where a primary study reported an outcome only for a propensity-matched or covariate-adjusted subset, that matched or adjusted estimate was not entered into the primary, full-cohort pooling; instead, it was reserved for a prespecified sensitivity analysis to avoid combining matched subsamples with full cohorts within the same pooled estimate. For two full-cohort outcomes reported only as an unadjusted odds ratio with a 95% confidence interval, without a tabulated 2 × 2 count (Jaquet et al. [18], 30-day mortality and pneumonia), the underlying cell counts were reconstructed algebraically from the reported odds ratio and confidence interval and entered as counts under the Mantel–Haenszel method. For one study with three extubation-timing subgroups (Malvindi et al. [21]), the ICE comparator for the primary analysis comprised all patients not extubated on-table (both the extubated-within-six-hours-in-ICU subgroup and the later, non-fast-track subgroup), giving an analyzed cohort of 160 ORE and 196 ICE patients, chosen to represent ICU extubation irrespective of timing rather than a fast-track subgroup alone. Because this ICU-extubated arm combines the fast-track (≤6 h) and non-fast-track (>6 h) subgroups, and the non-fast-track subgroup is partly defined by early adverse events, a prespecified leave-one-out analysis excluding Malvindi [21] was performed for the timing-sensitive endpoints (reintubation, reoperation for bleeding, and mortality). One denominator exception applies to new-onset atrial fibrillation: Malvindi [21] and Jaquet [18] reported this endpoint only among patients in preoperative sinus rhythm, so for this outcome Malvindi contributes 123 ORE and 139 ICE patients rather than its full 160 and 196, whereas Kruse [19] and Lim [20] reported all-comers; this non-comparability of denominators is the reason new-onset atrial fibrillation is treated as exploratory and is not presented as a headline pooled result (Section 3.3 and Section 4). The enrolled, matched, and analyzed sample sizes for each study and the single unit of analysis adopted for every pooled estimate, are detailed in Supplementary Table S1.

In addition to the primary, full-cohort analysis, a sensitivity analysis pooled confounding-adjusted estimates from the subset of studies reporting a propensity-matched or multivariable-adjusted result for a given endpoint (Jaquet et al. [18] and Kruse et al. [19]), using the generic inverse-variance method. Because individual patient data were not available across studies, propensity matching itself could not be performed at the meta-analytic level; this sensitivity analysis instead pools the adjusted estimates already computed within the primary studies. Prolonged mechanical ventilation, reported by two studies, was not analyzed as an outcome because, with extubation timing as the exposure, this endpoint is near zero in the ORE arm by construction and does not reflect a treatment effect. Length-of-stay and intraoperative time outcomes (ICU stay, hospital length of stay, cardiopulmonary bypass time, cross-clamp time) were not pooled as weighted mean differences given extreme between-study heterogeneity (*I*^2^ *=* 96 to 100 percent) and are instead summarized narratively.

## 3. Results

### 3.1. Baseline Characteristics

**Figure 1:**
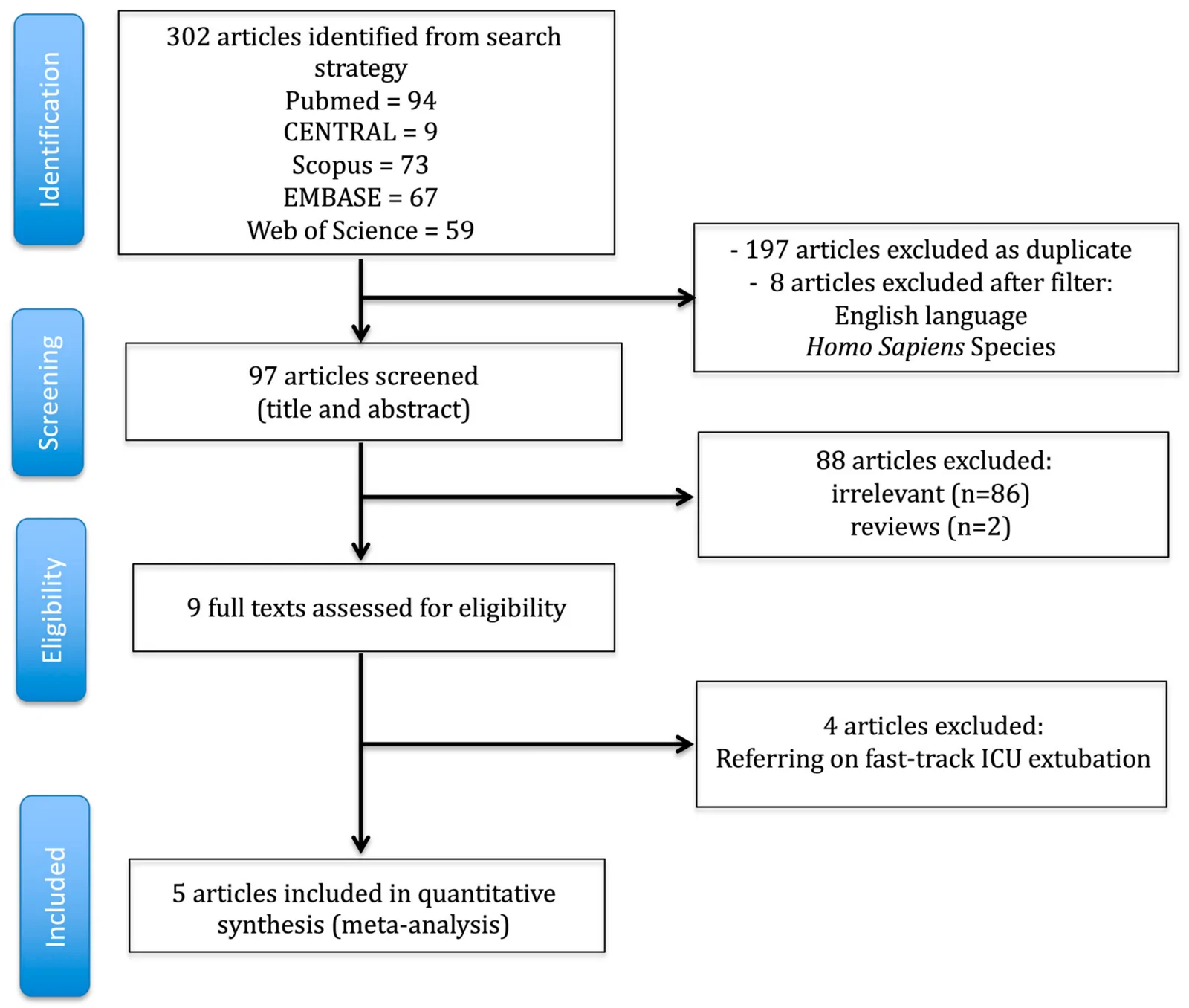
Trial flow diagram of the literature search and study selection process.

**Figure 2:**
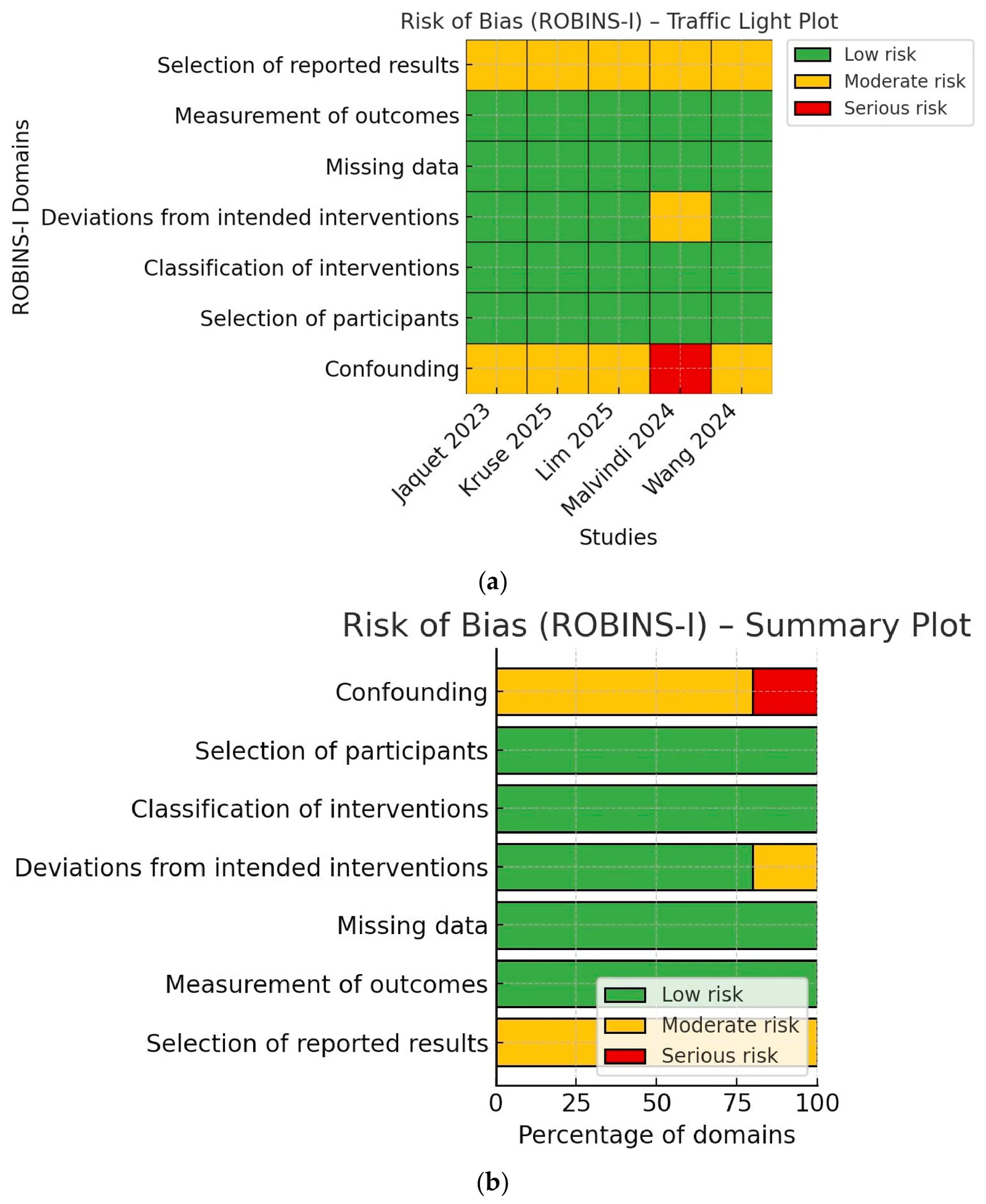
Risk of bias assessment using ROBINS-I. (**a**) Summary plot showing the proportion of each bias domain across included studies; (**b**) traffic light plot showing individual study-level risk of bias ratings.

The trial flow of the literature search is demonstrated in Figure 1. A total of 5 studies [18,19,20,21,22], published between 2023 and 2025, were incorporated, comprising 1101 patients extubated in the operating room and 899 extubated in the intensive care unit (*n* = 2000), applying a single unit of analysis as described in Section 2.4. The level of agreement was almost perfect (kappa = 0.83). The characteristics of the included studies are demonstrated in Table 1. The evaluation of the included studies according to the ROBINS-I criteria is demonstrated in Figure 2a,b. The reconciliation of enrolled, matched, and analyzed sample sizes across all five studies is provided in Supplementary Table S1, and the operating room extubation protocols, readiness criteria, and governance (institutional protocol versus individual anesthetist discretion) for each study are summarized in Supplementary Table S2.

Across the pooled cohorts, the proportion of female patients was similar between ORE and ICE (OR 0.93, 95% CI 0.69–1.26; *p* = 0.65; *I*^2^ = 54%). Rates of hypertension (OR 0.93, 95% CI 0.72–1.19; *p* = 0.54; *I*^2^ = 0%) and COPD (OR 0.83, 95% CI 0.52–1.32; *p* = 0.44; *I*^2^ = 7%) were comparable. Prior cerebrovascular accident (OR 0.75, 95% CI 0.42–1.37; *p* = 0.35; *I*^2^ = 50%) did not differ significantly between groups. Chronic kidney disease was less common in the ORE cohorts (OR 0.44, 95% CI 0.27–0.72; *p* < 0.01; *I*^2^ = 0%). This baseline imbalance indicates that patients with pre-existing renal impairment were preferentially retained intubated and extubated in the ICU and is direct evidence, discussed further in Section 4, that confounding by indication underlies several of the associations reported below.

Continuous baseline variables also demonstrated good comparability. Mean age did not differ significantly (WMD −1.79 years, 95% CI −3.91 to 0.33; *p* = 0.10), while BMI was also not significantly different (WMD −0.71 kg/m^2^, 95% CI −1.55 to 0.14; *p* = 0.10). Cardiopulmonary bypass and cross-clamp times were numerically shorter in the ORE group in most studies but were not pooled as weighted mean differences because between-study heterogeneity was extreme (*I*^2^ 97 to 100 percent), the same threshold applied to the length-of-stay outcomes; these operative times are therefore reported descriptively rather than as summary estimates. Rather than indicating balanced groups, this pattern of differences, most notably the lower prevalence of chronic kidney disease and the shorter bypass and cross-clamp times in the ORE arm, is consistent with preferential selection of lower-risk, more stable patients for operating room extubation and is considered further as confounding by indication in Section 4. A summary of pooled baseline and operative estimates is presented in Table 2.

### 3.2. Primary Endpoints

**Figure 3:**
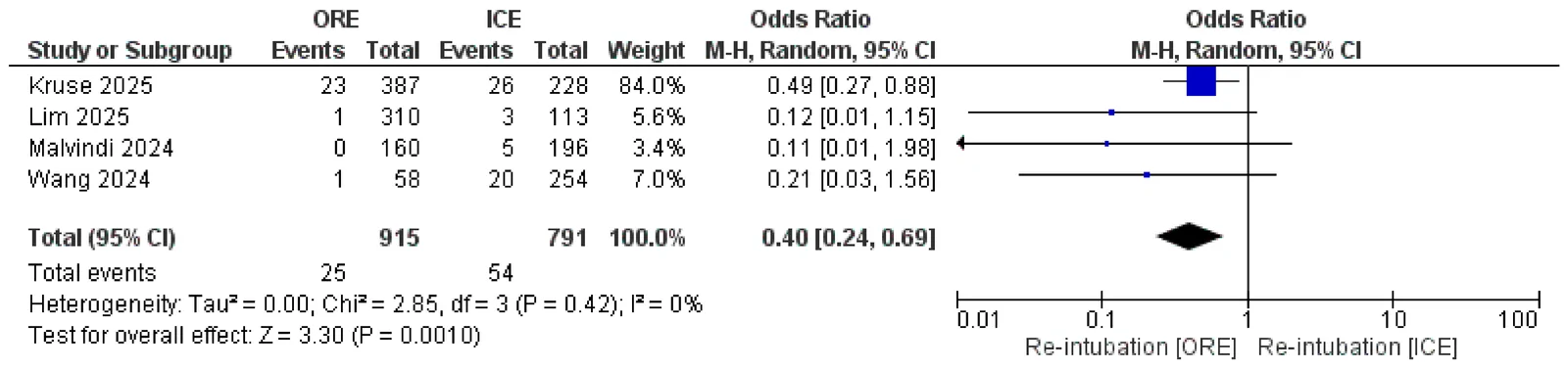
Forest plot comparing the odds of reintubation between operating room extubation (ORE) and intensive care unit extubation (ICE). M-H: Mantel–Haenszel; CI: confidence interval.

The primary endpoint of reintubation was reported in four studies (Kruse [19], Lim [20], Malvindi [21], Wang [22]); Jaquet [18] reported reintubation within a composite respiratory-complication outcome rather than as a standalone comparable cell and was not included in this pool. ORE was associated with significantly lower odds of reintubation compared with ICE (OR 0.40, 95% CI 0.24 to 0.69; *p* < 0.01; *n* = 915 ORE/791 ICE), with no observed heterogeneity (*I*^2^ = 0%) (Figure 3). In the prespecified analysis excluding Malvindi [21], the reintubation association was materially unchanged (OR 0.42, 95% CI 0.25 to 0.73; 3 studies; 755 ORE/595 ICE), as was the estimate for reoperation for bleeding (OR 0.52, 95% CI 0.17 to 1.59), indicating that the Malvindi [21] comparator was not the source of the observed associations. Consistent with the primary analysis, no pooled odds ratio is reported for 30-day mortality in this leave-one-out check.

### 3.3. Secondary Endpoints

**Figure 4:**
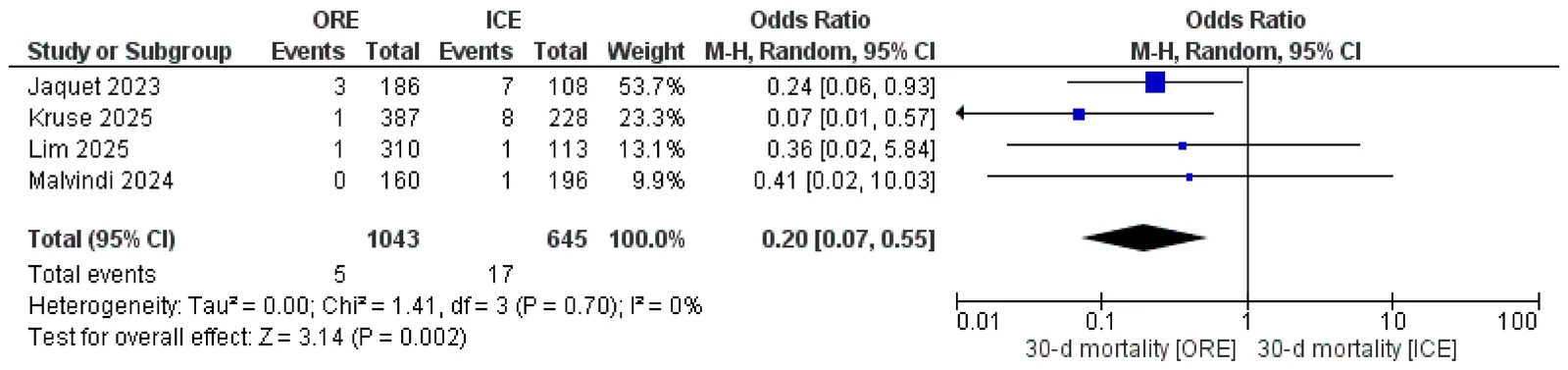
Forest plot comparing the odds of 30-day mortality between operating room extubation (ORE) and intensive care unit extubation (ICE) [18,19,20,21]. M-H: Mantel–Haenszel; CI: confidence interval. Individual study-level estimates are shown; no pooled odds ratio or overall-effect test is presented, and Wang 2024 (which reported no deaths in either arm) is not included for the reasons given in Section 3.3 and Section 4.

The odds of postoperative pneumonia were significantly lower after ORE. Pneumonia was reported in three studies (Jaquet [18], Kruse [19], Lim [20]), including one study (Jaquet [18]) whose full-cohort cell counts were reconstructed from its reported unadjusted odds ratio as described in Section 2.4: pooled OR 0.30, 95% CI 0.16 to 0.53; *p* < 0.001; *I*^2^ = 0% (*n* = 883 ORE/449 ICE). The incidence of postoperative delirium was significantly lower after ORE across four studies (OR 0.47, 95% CI 0.31 to 0.72; *p* < 0.001; *I*^2^ = 0%; *n* = 915 ORE/791 ICE). Rates of stroke were similar between groups across three studies (OR 0.54, 95% CI 0.17 to 1.70; *p* = 0.29; *I*^2^ = 0%; *n* = 857 ORE/537 ICE). New-onset atrial fibrillation occurred at numerically lower but not significantly different rates across three studies (OR 0.74, 95% CI 0.54 to 1.00; *p* = 0.05; *I*^2^ = 24%; *n* = 820 ORE/480 ICE). The denominators for this endpoint were not uniform across studies: Malvindi [21] and Jaquet [18] restricted new-onset atrial fibrillation to patients in preoperative sinus rhythm, whereas Kruse [19] and Lim [20] reported all-comers; because Malvindi [21] and Jaquet [18] restricted this endpoint to patients in preoperative sinus rhythm (Malvindi contributing 123 versus 139 rather than 160 versus 196), while Kruse [19] and Lim [20] reported all-comers, the pooled estimate combines non-comparable denominators; it is therefore reported as exploratory only, is not a headline result, and is not relied upon as evidence of confounding. Reoperation for bleeding did not differ significantly between groups once a single, full-cohort unit of analysis was applied across three studies (OR 0.40, 95% CI 0.16 to 1.01; *p* = 0.05; *I*^2^ = 45%; *n* = 857 ORE/537 ICE); this association was significant in an earlier, uncorrected pooling that entered one study at its matched subsample size, and its attenuation to non-significance in the full cohort is consistent with confounding by indication, as discussed in Section 4. Prolonged ventilation was not analyzed for the reasons given in Section 2.4. Thirty-day mortality was reported by four of the five included studies; Wang [22] did not report mortality and contributed no events to this analysis. Of the four reporting studies, two (Lim [20], 1 versus 1; Malvindi [21], 0 versus 1) were essentially uninformative, so the difference rests principally on Jaquet [18] (3 of 186 versus 7 of 108, reconstructed as described in Section 2.4 and cautioned by its own authors as likely attributable to chance) and Kruse [19] (1 of 387 versus 8 of 228, a pre- versus post-2021 protocol comparison subject to a secular era effect). Given the small number of events (5 of 1043 ORE versus 17 of 645 ICE across the four reporting studies), the concentration of deaths among the higher-risk patients preferentially retained intubated, and the confounded basis of the two informative cohorts, we do not report a pooled odds ratio and do not interpret this difference as a treatment effect of extubation location (Figure 4).

ICU stay and hospital length of stay were consistently shorter after ORE across the included studies; however, because between-study heterogeneity was extreme (*I*^2^ 96 to 100 percent) and two studies reported medians with interquartile ranges rather than means, these outcomes were not pooled as weighted mean differences and are not summarized by a single estimate. A summary of the pooled binary-outcome estimates is presented in Table 3.

### 3.4. Publication Bias and Heterogeneity

Heterogeneity was low for the primary reintubation endpoint and for most secondary binary endpoints (*I*^2^ 0 to 45 percent) and remained extreme for the continuous outcomes, which were therefore not pooled (Section 3.3). With fewer than ten studies contributing to any endpoint, funnel-plot methods and formal tests for small-study effects were not used, and publication or small-study bias could not be assessed for any endpoint. A leave-one-out sensitivity analysis was performed for all pooled endpoints and did not materially change the direction or significance of the primary estimates.

### 3.5. Sensitivity Analysis Using Confounding-Adjusted Estimates

To address the possibility that the primary associations reflect confounding by indication rather than a treatment effect, we separately pooled the propensity-matched or covariate-adjusted estimates reported by the primary studies using the generic inverse-variance method. Two studies contributed adjusted or matched estimates for at least one endpoint (Jaquet [18] and Kruse [19]); a third (Malvindi [21]) reported adjusted estimates only for length-of-stay outcomes, which were not pooled for the reasons given in Section 3.3.

Reintubation attenuated toward the null when pooling Kruse’s [19] matched cohort and Jaquet’s [18] adjusted odds ratio (adjusted OR 0.47, 95% CI: 0.22 to 1.00; *I*^2^ = 0%; 2 studies). For 30-day mortality, the only adjusted or matched estimate available was the propensity-matched cohort of Kruse [19], in which the operating room arm contained zero deaths (0 of 122 versus 5 of 122); any matched odds ratio for this endpoint therefore rests on a zero-cell continuity correction and is not reported, consistent with the primary analysis (Section 3.3). Pneumonia showed a similar point estimate to the primary analysis but with a confidence interval crossing unity and moderate heterogeneity between the two contributing adjusted estimates (adjusted OR 0.32, 95% CI: 0.07 to 1.51; *I*^2^ = 54%; 2 studies); the larger adjustment reported by Jaquet [18] relative to Kruse’s [19] matched estimate suggests that residual, unmeasured confounding may act in the same direction as the observed association rather than against it. New-onset atrial fibrillation likewise moved from an apparent benefit in the crude analysis to a null adjusted estimate; as noted in Section 3.3; however, its non-uniform denominators limit the weight that can be placed on this shift, and it is reported here for completeness rather than as primary evidence (adjusted OR 1.13, 95% CI: 0.75 to 1.70; *I*^2^ = 0%; 2 studies), with the point estimate crossing to the opposite side of unity from the primary crude estimate. Delirium, reoperation for bleeding, and stroke were each informed by only a single study reporting a matched estimate and are presented in Table 4 as exploratory checks rather than pooled results; reoperation for bleeding in particular has no independent adjusted corroboration and should be interpreted with corresponding caution. Taken together, this sensitivity analysis indicates that a substantial portion of the associations observed in the primary analysis, most clearly for reoperation for bleeding, is attributable to confounding by indication rather than a causal effect of extubation location. A summary is presented in Table 4.

## 4. Discussion

The present systematic review and meta-analysis synthesizes available evidence comparing extubation in the operating room versus the intensive care unit after minimally invasive cardiac valve surgery. Across 2000 patients from five contemporary cohorts, applying a single, consistent unit of analysis, OR extubation was associated with lower odds of reintubation, postoperative delirium, and pneumonia, with no significant difference in stroke, new-onset atrial fibrillation, or reoperation for bleeding, and with shorter ICU and hospital stays that could not be formally pooled. A lower pooled mortality was also observed. As detailed below, we interpret these associations principally as evidence of patient selection rather than as a demonstrated causal benefit of extubation location.

Confounding by indication is the central methodological consideration in interpreting these results. In every included cohort, ORE was assigned to patients already demonstrating intraoperative and early postoperative stability, including short bypass times, adequate gas exchange, minimal chest-tube output, and low inotrope requirement. The significant baseline imbalance in chronic kidney disease (OR: 0.44, *p* < 0.01) documented in Section 3.1 is direct evidence that higher-risk patients were preferentially retained intubated. This has two consequences for interpretation. First, endpoints that are themselves close correlates of the selection criteria, most clearly reoperation for bleeding, are expected to appear favorable for ORE largely because of reverse causation (a patient who is actively bleeding is, by definition, not extubated on the table) rather than because early extubation prevents bleeding. This interpretation is reinforced by the primary studies themselves: several explicitly list the absence of bleeding as a criterion for on-table extubation, and Wang [22] and Lim [20] state that bleeding-prone patients were excluded from operating room extubation. The pooled reoperation-for-bleeding estimate also combines event definitions of differing severity, since a takeback triggered by persistent chest-tube output captures earlier and less severe events than one encoding a stricter tamponade- or shock-driven threshold, which may contribute to the moderate heterogeneity observed for this endpoint. Consistent with this, reoperation for bleeding lost statistical significance once a single, full-cohort unit of analysis was applied (Section 3.3), and its adjusted, single-study estimate remains wide and uncertain. Second, the low statistical heterogeneity observed for most binary endpoints (*I*^2^ 0 to 45 percent) should not be read as evidence of robustness. Because the direction of confounding is shared across the cohorts, with the ICU-extubated arm being the older, higher-risk, or failure-selected group in every study, pooling concentrates rather than cancels this bias, and the studies agree in part because they share it. Moreover, three of the five cohorts are effectively era comparisons (Kruse [19], a pre- versus post-2021 protocol change; Wang [22], an interrupted time series; and Lim [20], in which on-table extubation reached 96.6 percent by 2023), so a portion of every association reflects secular improvement in perioperative care between approximately 2016 and 2023 rather than extubation location itself. Taken together with the reverse-causal nature of reoperation for bleeding, these considerations indicate that extubation timing functions as a surrogate marker for early postoperative stability rather than as an independent determinant of the outcomes examined, and we have accordingly revised our interpretation from causal, practice-change language to associational and feasibility language throughout.

The benefits observed in this analysis align with the theoretical rationale underlying fast-track cardiac anesthesia programs. Extubation in the operating room minimizes exposure to sedatives, allows earlier mobilization, and reduces the physiological stress associated with prolonged positive-pressure ventilation. Prior studies in conventional sternotomy cardiac surgery have demonstrated similar advantages of expedited extubation, including reductions in ICU resource utilization, pulmonary complications, and costs [23,24]. Importantly, minimally invasive valve surgery, characterized by lower surgical trauma, reduced postoperative pain, and shorter cardiopulmonary bypass times, provides a setting in which early extubation may be more readily achieved in appropriately selected patients [1,2,3,4,10,11].

The benefits observed for reintubation, delirium, and pneumonia are more plausibly attributable, at least in part, to a genuine effect of extubation strategy, since these endpoints are less directly definitional consequences of the selection criteria than reoperation for bleeding. Reintubation after cardiac surgery is associated with substantial increases in mortality, prolonged ventilation, and ICU burden [25]. Delirium, a complication known to prolong hospitalization and worsen long-term cognitive outcomes, was also lower among OR-extubated patients, consistent with evidence that shorter mechanical ventilation and reduced sedative exposure lower delirium risk in cardiac surgical populations [26,27]. Nonetheless, the sensitivity analysis showed that both associations attenuated somewhat when adjusted estimates were pooled (reintubation adjusted OR 0.47 versus primary OR 0.40), indicating that confounding by indication likely contributes to, without fully accounting for, these associations. The absolute event rate for reintubation in the ORE group was low (25 of 915 patients across the four reporting studies); this reinforces the feasibility of early extubation within carefully structured perioperative pathways but should be interpreted in light of the lower-risk phenotype that was selected for ORE in every included study rather than as evidence of a uniformly protective effect of extubation location.

As noted above, reoperation for bleeding is the clearest example of an endpoint that is a close correlate of the ORE selection criteria rather than an independent outcome: patients demonstrating early hemodynamic stability, minimal chest-tube output, and appropriate coagulation parameters are preferentially selected for OR extubation, and a patient who is actively bleeding is, almost by definition, not extubated on the table. This supports the concept that extubation timing acts as a surrogate marker for early postoperative stability rather than as an independent therapeutic intervention for this endpoint [28].

Length-of-stay outcomes are of clear clinical interest, but their between-study heterogeneity in the present analysis was too extreme to support a pooled summary estimate (Section 3.3), and we therefore do not report a single pooled reduction in ICU or hospital stay. Across the individual studies, both ICU and hospital stays were consistently shorter after operating room extubation, and prior ERAS and ERACS literature likewise identifies early extubation as an important driver of length-of-stay reduction after cardiac surgery [8,9]. Given persistent ICU bed shortages and the cost of postoperative critical care, a genuine reduction in stay would be clinically meaningful for patient flow and institutional efficiency; however, in view of the observational design and the selection of lower-risk patients for operating room extubation, the direction and magnitude of any such reduction require prospective confirmation.

Thirty-day mortality warrants particular caution in interpretation, and for this reason no pooled odds ratio is reported for this endpoint (Section 3.3). Mortality is the endpoint most susceptible to confounding by indication in this dataset: patients extubated in the ICU were, across every included study, the higher-risk patients who were not considered candidates for immediate extubation, whether because of bleeding, haemodynamic instability, longer bypass times, or comorbidity burden. The sensitivity analysis using adjusted estimates supported a protective association of similar direction, but the small number of events (5 ORE deaths and 17 ICE deaths across 2000 patients in the primary analysis) makes this estimate fragile, and we do not interpret it as evidence that the location of extubation itself reduces mortality. In the included cohorts, patients extubated in the OR were characterized as hemodynamically stable, normothermic, and exhibiting adequate gas exchange, criteria that align with international guidelines for fast-track extubation [12]. Early extubation strategies more broadly have been associated with low morbidity in prior single-center and registry-based series [29,30,31], although, as with the present analysis, those studies share the same vulnerability to confounding by indication and do not establish a causal benefit of extubation timing itself. Given the confounding-by-indication framework outlined above, we consider these criteria not incidental descriptors but the mechanism by which the observed associations arise: extubation location is largely a marker of, rather than an independent cause of, the favorable recovery profile.

These findings support the feasibility of operating room extubation, implemented within a structured fast-track anesthesia framework and with explicit, protocolized selection criteria, in appropriately selected patients undergoing minimally invasive valve surgery. Because the available evidence is entirely observational and substantially shaped by patient selection, we do not consider these data sufficient to recommend a specific practice change or to support unselected wider adoption of OR extubation; rather, they motivate protocolized, prospective evaluation of standardized readiness criteria (hemodynamic stability, minimal inotropic requirement, normothermia, adequate lung mechanics) as a basis for future comparative or randomized studies.

Several limitations merit consideration. First, all included studies were observational, and, as discussed above, confounding by indication substantially shapes several of the reported associations; this limits causal inference for every endpoint and precludes it entirely for endpoints, such as reoperation for bleeding, that are close correlates of the selection criteria themselves. Second, clinical heterogeneity existed across studies in surgical procedure mix (mitral versus aortic), anesthetic regimen, and the specific extubation readiness criteria applied; this heterogeneity, distinct from the statistical heterogeneity discussed for continuous outcomes, further constrains the strength of inference that can be drawn from the pooled binary endpoints. Third, governance of the extubation decision differed across studies, from a standardized institutional protocol in four cohorts to individual anesthetist discretion in one (Jaquet [18]), which may itself be a source of unmeasured variability; these protocol and readiness differences are documented per study in Supplementary Table S2. Fourth, with fewer than ten studies per endpoint, publication and small-study bias could not be formally assessed. Fifth, endpoints informed by few studies yield less stable between-study variance estimates under a random-effects model and should be interpreted cautiously; this applies in particular to the pneumonia estimate in the sensitivity analysis (two contributing estimates) and to the single-study exploratory rows in Table 4 (delirium, reoperation for bleeding, and stroke). Sixth, data on long-term outcomes, such as readmissions, long-term neurocognitive function, and quality of life, were scarce. Finally, continuous outcomes, particularly ICU and hospital length of stay, demonstrated heterogeneity too extreme to summarize with a single pooled estimate, reflecting differences in institutional pathways, ICU staffing models, and discharge practices.

Future research should prioritize prospective multicenter studies or randomized trials within ERACS frameworks to clearly delineate causality and identify which patient subgroups derive the greatest benefit from OR extubation. Incorporation of standardized extubation checklists, objective readiness scores, and refined analgesic pathways may further improve safety and expand eligibility. Additionally, economic analyses are needed to determine the cost savings achievable through routine OR extubation after minimally invasive valve surgery.

In summary, this systematic review and meta-analysis indicates that extubation in the operating room following minimally invasive cardiac valve surgery is associated with a favorable postoperative recovery profile for several endpoints, most plausibly reintubation, delirium, and pneumonia, without evidence of increased harm. However, because extubation location in every included study was determined by patient selection rather than randomization, and because reoperation for bleeding, the endpoint most closely tied to the selection criteria, lost its apparent benefit once a single, full-cohort unit of analysis was applied, these findings should be read as supporting the feasibility of OR extubation in carefully selected patients rather than as establishing a causal benefit of the strategy itself.

## 5. Conclusions

In this meta-analysis of five observational cohorts, extubation in the operating room after minimally invasive cardiac valve surgery was feasible and was associated with lower rates of reintubation, postoperative delirium, and pneumonia, without a signal of increased harm. These associations are, however, substantially shaped by confounding by indication: operating room extubation was offered to patients already demonstrating intraoperative and early postoperative stability, so several endpoints, most clearly reoperation for bleeding, partly or fully restate the selection rule rather than measure an independent consequence of extubation location. Extubation timing is therefore best understood, on the basis of the present evidence, as a marker of early postoperative stability rather than as a demonstrated therapeutic intervention in its own right. As minimally invasive valve surgery continues to expand, prospective and ideally randomized studies with prespecified, protocolized selection criteria are needed to determine whether operating room extubation confers benefit beyond patient selection and to establish standardized readiness assessments before this strategy can be recommended for broader adoption.

## Figures and Tables

**Table 1:** Baseline characteristics of the included studies. NOS score: Newcastle–Ottawa Scale rating (0–9) as assessed in the present meta-analysis; AVR: aortic valve replacement; MV: mitral valve; ORE: operating room extubation; ICE: intensive care unit extubation.

Study (Year)	Country	Study Design	Study Period	Population/ Approach	Sample Size	Extubation Groups	NOS Score
Jaquet 2023 [18]	Belgium	Retrospective observational	2016–2021	AVR, MV surgery	294 total	186 ORE/108 ICE	7/9
Kruse 2025 [19]	Germany	Retrospective cohort	2019–2023	AVR, MV surgery	615 total	387 ORE/228 ICE	8/9
Lim 2025 [20]	South Korea	Retrospective cohort	2012–2023	AVR	423 total	310 ORE/113 ICE	8/9
Malvindi 2024 [21]	Italy	Retrospective cohort	2018–2023	MV surgery	356 total	160 ORE/196 ICE	8/9
Wang 2024 [22]	United States	Retrospective chart review	2017–2022	AVR, MV surgery	312 total	58 ORE/254 ICE	7/9

**Table 2:** Summary of the pooled estimates of the baseline and operative characteristics of the included patients. HTN: Hypertension; COPD: Chronic Obstructive Pulmonary Disease; CVA: Cerebrovascular Accident; CKD: Chronic Kidney Disease; BMI: Body Mass Index; CPB: Cardiopulmonary Bypass; n: number; OR: Odds Ratio; WMD: Weighted Mean Difference; 95% CI: 95% Confidence Intervals.

Outcome	*n*	OR or WMD [95% CI]	*p*	*I*^2^ (%)	*p*-Het
Categorical Outcomes					
Females	5	OR 0.93 [0.69, 1.26]	0.65	54%	0.07
HTN	3	OR 0.93 [0.72, 1.19]	0.54	0%	0.76
COPD	4	OR 0.83 [0.52, 1.32]	0.44	7%	0.36
CVA	3	OR 0.75 [0.42, 1.37]	0.35	50%	0.14
CKD	3	OR 0.44 [0.27, 0.72]	<0.01	0%	0.55
Continuous Outcomes					
Age (years)	5	WMD −1.79 [−3.91, 0.33]	0.10	93%	<0.05
BMI (kg/m^2^)	4	WMD −0.71 [−1.55, 0.14]	0.10	71%	0.01
CPB time (min)	4	WMD −11.78 [−22.70, −0.85]	0.03	97%	<0.01
Cross-clamp time (min)	4	WMD −18.24 [−46.92, 10.44]	0.21	100%	<0.01

**Table 3:** Summary of the pooled estimates of postoperative outcomes. AF: Atrial Fibrillation; LOS: Length of Stay; n: number; OR: Odds Ratio; WMD: Weighted Mean Difference; 95% CI: 95% Confidence Intervals. p-het: *p*-value for between-study heterogeneity. For 30-day mortality and pneumonia, the cell counts for Jaquet et al. [18] were reconstructed from the reported crude (unadjusted) odds ratio and its 95% confidence interval; no zero-cell continuity correction was applied to the source estimate. A pooled odds ratio is not reported for 30-day mortality; raw events across the four studies reporting this outcome are shown instead for the reasons detailed in Section 3.3. The new-onset atrial fibrillation estimate is exploratory and is not a headline result because two studies restricted this endpoint to patients in preoperative sinus rhythm while two reported all-comers, giving non-comparable denominators (Section 2.4 and Section 3.3).

Outcome	*n*	OR or WMD [95% CI]	*p*	*I*^2^ (%)	*p*-Het
Categorical Outcomes					
Pneumonia	3	0.30 [0.16, 0.53]	<0.001	0%	0.37
Re-intubation	4	0.40 [0.24, 0.69]	<0.001	0%	0.42
Reoperation for Bleeding	3	0.40 [0.16, 1.01]	0.05	45%	0.16
Delirium	4	0.47 [0.31, 0.72]	<0.001	0%	0.77
New AF (exploratory)	3	0.74 [0.54, 1.00]	0.05	24%	0.12
Stroke	3	0.54 [0.17, 1.70]	0.29	0%	0.60
30-day Mortality	4	Not pooled; raw events 5/1043 vs. 17/645	n/a	n/a	n/a

**Table 4:** Sensitivity analysis pooling confounding-adjusted estimates (generic inverse-variance method). AF: atrial fibrillation. New table, added in revision. For 30-day mortality, the only matched estimate available (Kruse [19]) contained zero deaths in the operating room arm (0 of 122 versus 5 of 122); any odds ratio for this row would depend entirely on an arbitrary zero-cell continuity correction, so no adjusted odds ratio is reported for mortality, consistent with the primary analysis (Section 3.3 and Section 3.5).

Outcome	Studies	Adjusted OR [95% CI]	*I* ^2^	Basis
Reintubation	2	0.47 [0.22, 1.00]	0%	Kruse [19] (matched), Jaquet [18] (adjusted OR)
30-day mortality	2	Not reported (zero-cell)	n/a	Kruse [19] (matched, 0 of 122 vs. 5 of 122) and Jaquet [18] (adjusted OR); not pooled owing to zero-cell (see footnote)
Pneumonia	2	0.32 [0.07, 1.51]	54%	Kruse [19] (matched), Jaquet [18] (adjusted OR)
New-onset AF	2	1.13 [0.75, 1.70]	0%	Kruse [19] (matched), Jaquet [18] (adjusted OR)
Delirium	1	0.66 [0.29, 1.48]	n/a	Kruse [19] (matched); exploratory, not pooled
Reoperation for bleeding	1	0.11 [0.01, 2.02]	n/a	Kruse [19] (matched); exploratory, not pooled
Stroke	1	0.74 [0.16, 3.40]	n/a	Kruse [19] (matched); exploratory, not pooled

## Data Availability

All raw data are available upon reasonable request.

## Supplementary Materials

The following supporting information can be downloaded at https://www.mdpi.com/article/10.3390/jcdd13080368/s1, Table S1. Unit-of-analysis reconciliation across the five included studies; Table S2. Operating-room/on-table extubation protocols and readiness criteria in the included studies.

## Abbreviations

The following abbreviations are used in this manuscript:

AVR	Aortic Valve Replacement
CI	Confidence Interval
CPB	Cardiopulmonary Bypass
ERAS	Enhanced Recovery After Surgery
ERACS	Enhanced Recovery After Cardiac Surgery
ICE	Intensive Care Unit Extubation
ICU	Intensive Care Unit
*I*^2^	Inconsistency Index
LOS	Length of Stay
MV	Mitral Valve
NOS	Newcastle–Ottawa Scale
OR	Odds Ratio
ORE	Operating Room Extubation
PRISMA	Preferred Reporting Items for Systematic Reviews and Meta-Analyses
ROBINS-I	Risk of Bias in Non-Randomized Studies of Interventions
WMD	Weighted Mean Difference
