# Supplementary material for: Feasibility and Safety of Operating Room Extubation After Minimally Invasive Cardiac Valve Surgery: A Systematic Review and Meta-Analysis
Source: J Cardiovasc Dev Dis. 2026 Aug 4;13(8):368. doi: 10.3390/jcdd13080368 (PMC13513249; doi:10.3390/jcdd13080368)
Supplement: Supplementary file 1 [file jcdd-13-00368-s001.zip › jcdd-4371460-supplementary.pdf]

Table S1. Unit-of-analysis reconciliation across the five included studies.

This table documents the single unit of analysis adopted for the meta-analysis and reconciles the enrolled, matched, and analyzed sample sizes for each study. An earlier headline enrollment (836 ORE / 719 ICE; n = 1,555) arose from pooling Kruse at its matched size (122 / 122) together with the remaining studies at full-cohort or subgroup size, while the baseline comparison used full cohorts. In the present analysis every study contributes its full reported cohort, giving 1,101 ORE and 899 ICE patients (n = 2,000); matched or adjusted estimates from Jaquet and Kruse are reserved for the sensitivity analysis (Table 4 of the main text).

Study (year) [ref]	Design and OR-extubation governance	Enrolled cohort, ORE / ICE (n)	Matched / adjusted subset reported	N that entered earlier headline (836 / 719)	Recommended primary-analysis N (full cohort)
**Jaquet 2023 [18]**	Single-center retrospective; on-table extubation practiced by 3 of 6 anesthesiologists (individual discretion, not policy)	186 / 108 (n = 294)	Yes: 1:1 propensity-matched 76 / 76; plus multivariable-adjusted ORs for all secondary outcomes	186 / 108 (full) in header, BUT 76 / 76 (matched) in the mortality forest plot	186 / 108
**Kruse 2025 [19]**	Single-center retrospective; institutional ultra-fast-track protocol, standard of care since 2021	387 / 228 (n = 615)	Yes: 1:1 case-control matched 122 / 122 (age, BMI, cross-clamp)	122 / 122 (matched) -- the source of the mismatch	387 / 228
**Lim 2025 [20]**	Single-center retrospective; institutional routine since 2012 (about 96.6% by 2023)	310 / 113 (n = 423)	No matched subset (multivariable used only to predict extubation success, not outcomes)	310 / 113 (full)	310 / 113
**Malvindi 2024 [21]**	Single-center retrospective; institutional ultra-fast-track protocol since 2016	160 on-table / 122 ICU-<=6h / 74 non-fast-track >6h (n = 356)	No matched subset; adjusted ORs for LOS endpoints only	160 / 122 (on-table vs ICU-<=6h; the 74 >6h patients dropped)	160 / 196 (ICE = all patients not extubated on-table, i.e. the <=6 h and >6 h ICU subgroups combined)
**Wang 2024 [22]**	Single-center retrospective chart review; institutional protocol implemented 20 Aug 2020 (interrupted time series)	58 / 254 (n = 312)	No matched subset	58 / 254 (full)	58 / 254
**TOTAL**		**1,101 / 899 (2,000)**		**836 / 719 (1,555)**	**1,101 / 899 (2,000)**

Table S2. Operating-room / on-table extubation protocols and readiness criteria in the included studies.

Criteria are reported as stated in each source. Governance distinguishes a standardized institutional protocol from extubation left to individual anesthetist judgment (relevant to Reviewer 2, comments 1 and 2). RAMT: right anterior mini-thoracotomy; AVR: aortic valve replacement; CPB: cardiopulmonary bypass; TOF: train-of-four; EMG: electromyography.

Study (year) [ref]	Governance: institutional protocol vs individual discretion	OR / on-table extubation readiness criteria (as reported)	Neuromuscular reversal and monitoring
**Jaquet 2023 [18]**	Individual anesthetist discretion. Only 3 of 6 cardiac anesthesiologists practiced on-table extubation; patients randomly assigned to an anesthesiologist.	Spontaneous breathing with respiratory rate >10/min and tidal volume >6 mL/kg; oriented response to verbal command; blood loss <200 mL since skin closure; full recovery from neuromuscular blockade.	Train-of-four ratio >0.9 required before extubation.
**Kruse 2025 [19]**	Institutional protocol, standardized department policy since 2021; became standard of care (pre- vs post-2021 time-based comparison).	Adequate warmth; stable gas exchange; sufficient coagulation and hemostasis; minimal bleeding; controlled vasopressors.	Rocuronium-based; standardized anesthesia. Reversal not separately specified in protocol figure.
**Lim 2025 [20]**	Institutional; routine since 2012, applied to all RAMT-isolated AVR candidates, rising to about 96.6% by 2023.	No bleeding; stable vital signs; neurologically intact (return of consciousness, cough and gag reflex); adequate oxygenation SpO2 >95% at FiO2 <=0.4; normothermia >36 C; tidal volume >5 mL/kg; respiratory rate 10 to 25/min; low-dose inotrope/vasopressor permitted (dobutamine <=5 mcg/kg/min, norepinephrine <=0.1 mcg/kg/min); no significant post-CPB arrhythmia.	Sugammadex after skin closure; quantitative EMG monitor (TwitchView); TOF ratio >0.9 confirmed.
**Malvindi 2024 [21]**	Institutional ultra-fast-track protocol since 2016, applied uniformly to all transaxillary mitral cases.	Patient alert, awake, able to follow commands; hemodynamic stability without inotropic support; no bleeding (surgeon-assessed during and after closure); hemoglobin >10 g/L; body temperature >36 C; spontaneous breathing with respiratory rate <30/min; appropriate acid-base status; PaCO2 <50 mmHg, PaO2 >60 mmHg at FiO2 <=50%; SpO2 >95%; tidal volume 5 to 10 mL/kg.	Low-dose opioid-based anesthesia with short-acting volatile agent (sevoflurane); reversal not separately specified.
**Wang 2024 [22]**	Institutional protocol implemented 20 Aug 2020 (interrupted time series, early vs late era).	Institutional guideline (Supplementary Fig 2): age <65 years; BMI <35 kg/m2; absence of severe lung disease; intraoperative clinician judgment. Shorter bypass length and lower intraoperative opioid dose were independently associated with successful OR extubation.	Erector spinae blocks used widely from Jan 2019; specific reversal protocol not detailed in main text.
